# Natural Language Processing in aid of FlyBase curators

**DOI:** 10.1186/1471-2105-9-193

**Published:** 2008-04-14

**Authors:** Nikiforos Karamanis, Ruth Seal, Ian Lewin, Peter McQuilton, Andreas Vlachos, Caroline Gasperin, Rachel Drysdale, Ted Briscoe

**Affiliations:** 1 Computer Laboratory, University of Cambridge, William Gates Building, Cambridge, CB3 0FD, UK; 2 Department of Genetics, University of Cambridge, Downing Site, Cambridge, CB2 3EH, UK

## Abstract

**Background:**

Despite increasing interest in applying Natural Language Processing (NLP) to biomedical text, whether this technology can facilitate tasks such as database curation remains unclear.

**Results:**

PaperBrowser is the first NLP-powered interface that was developed under a user-centered approach to improve the way in which FlyBase curators navigate an article. In this paper, we first discuss how observing curators at work informed the design and evaluation of PaperBrowser. Then, we present how we appraise PaperBrowser's navigational functionalities in a user-based study using a text highlighting task and evaluation criteria of Human-Computer Interaction. Our results show that PaperBrowser reduces the amount of interactions between two highlighting events and therefore improves navigational efficiency by about 58% compared to the navigational mechanism that was previously available to the curators. Moreover, PaperBrowser is shown to provide curators with enhanced navigational utility by over 74% irrespective of the different ways in which they highlight text in the article.

**Conclusion:**

We show that state-of-the-art performance in certain NLP tasks such as Named Entity Recognition and Anaphora Resolution can be combined with the navigational functionalities of PaperBrowser to support curation quite successfully.

## Background

Due to the enormous growth of literature in biosciences, several research groups have been developing search engines with more advanced functionalities than PubMed and Google (see [1] for an overview). MedMiner [2], Textpresso [3], iHop [4] and EBIMed [5] are characteristic examples. Such systems are primarily designed to perform *information retrieval*, i.e. to return documents relevant to a query within a large collection. This is typically accomplished without incorporating advanced Natural Language Processing (NLP) techniques such as those discussed in [6]. With the exception of the BioText Search Engine [7], most of these systems are not reported to have been developed by soliciting input from their intended users.

As Cohen et al. [8] assert, the time has now come to start exploring the usability of biomedical text mining tools, including systems which use advanced NLP techniques for tasks such as Named Entity Recognition (NER) and Anaphora Resolution. Professional curators, who are responsible for populating databases with information derived from the literature, are among the intended users of these systems. However, as concluded in the most recent exploration of this issue by Alex et al. [9], whether advanced NLP technology can facilitate database curation remains unclear.

PaperBrowser [10] is the first NLP-powered curation interface to be developed under a user-centered approach. It was integrated within a curation workflow to improve the way in which curators navigate an article. This paper presents the most recent version of PaperBrowser and discusses how we assessed it by applying evaluation criteria of Human-Computer Interaction (HCI). Our results show that PaperBrowser improves navigational efficiency by about 58% and provides curators with enhanced utility by over 74% compared to the navigational mechanism that was previously available to them.

The paper is organised as follows: First, we discuss how observing curators at work informed the design and evaluation of PaperBrowser. Then, we present its main functionalities for indexing an article and outline the NLP processes that underlie them. The rest of the paper is devoted to our methods for appraising PaperBrowser as a navigational aid for curation.

## Implementation

### Observing curators at work

PaperBrowser has been developed under a user-centered approach, in which the intended user is actively involved in every phase of software development [11]. Its aim is to assist the FlyBase literature curation team in Cambridge, which currently consists of five full-time curators. FlyBase is a widely used database of genomic research on the fruit fly [12]. Founded in 1992, FlyBase is updated with information from literature, other biological databases and the research community by curation teams located in three different sites. Since the curation paradigm followed by FlyBase has been adopted by several other curation groups, interest in our efforts is not restricted to the FlyBase domain but extends to the wider curation community.

FlyBase literature curation is based on a watchlist of around 35 journals. Each curator routinely selects a journal from the list and inspects its latest issue to identify which articles to curate. Curation takes place on an article-by-article basis (as opposed to gene-by-gene or topic-by-topic). In our previous work [10], we outlined the curation workflow and discussed why extant information retrieval tools which are devised to support the topic-by-topic curation model do not support the article-by-article curation paradigm followed by FlyBase. In this section, we discuss how observing curators informed our system design.

In accordance with the user-centered model for system development, we observed curators at work to gain insight into their practices. Two curators were observed by the first author who was keeping notes. The observations took place in the curators' office and lasted for six working days (3 days per curator).

The observer focused on the way in which the curators interact with the curated article, which is typically viewed in printed form or online (as PDF or HTML). We noticed that curators do not read the article from the beginning to the end. Rather, they look for regions in the article which contain a lot of curatable information. Once such a region is identified, they highlight segments of text within that region on their hard copy with their marker. Afterwards, they look further away in the article for another curatable region. The "Find" function is often used to search the electronic version of the article for multiple occurrences of the same term and identify a curatable region. Since curatable entities are often expressed by various names which may also correspond to common English words [13], the result of this operation contains a lot of noise that hinders curation.

Similar information is usually conveyed in various parts of the article, often several pages away, and curators need to compare these excerpts with each other to decide exactly what will be curated. Using "Find" to "flip back and forth" between the different parts of the article and compare them adds significantly to the curation effort.

The exact way in which the curators navigate the article using "Find" varies from one individual to the other. Curators also differ in their *highlighting density*, i.e. the way in which they highlight text: Once a single segment that contains several chunks of curatable information is detected, some curators might mark it with just a few actions (low highlighting density). Other curators prefer to perform more fine-grained highlighting within the segment, one for each curatable chunk of information (high highlighting density). The sectioning of the document as well as the names of genes and related entities which are mentioned in it are known to be important clues for curation [14]. However, our observations led us to the conclusion that neither the article printout nor its online version enable the curators to make adequate use of these clues. Thus, our first aim was to develop NLP technology which can identify such curation clues relatively reliably. Additionally, the insights from our observations were used to develop an interface which exploits the NLP analysis to provide the curators with enhanced modes of navigating the text. Evaluation criteria from HCI were applied to assess this interface.

An alternative option would have been to build a traditional information extraction system such as the one discussed by [15] that would provide the user with automatically filled templates. However, as already argued by [1], this would not have been particularly helpful since it shifts the curators' responsibility to verifying the quality of the NLP output, often forcing them to go back to the text and confirm its validity. Instead, we set out to develop a system in which the users maintain the initiative following their preferred curation style but are usefully assisted by software adapted to their work practices. This system is described in the next section.

### PaperBrowser

**Figure 1:**
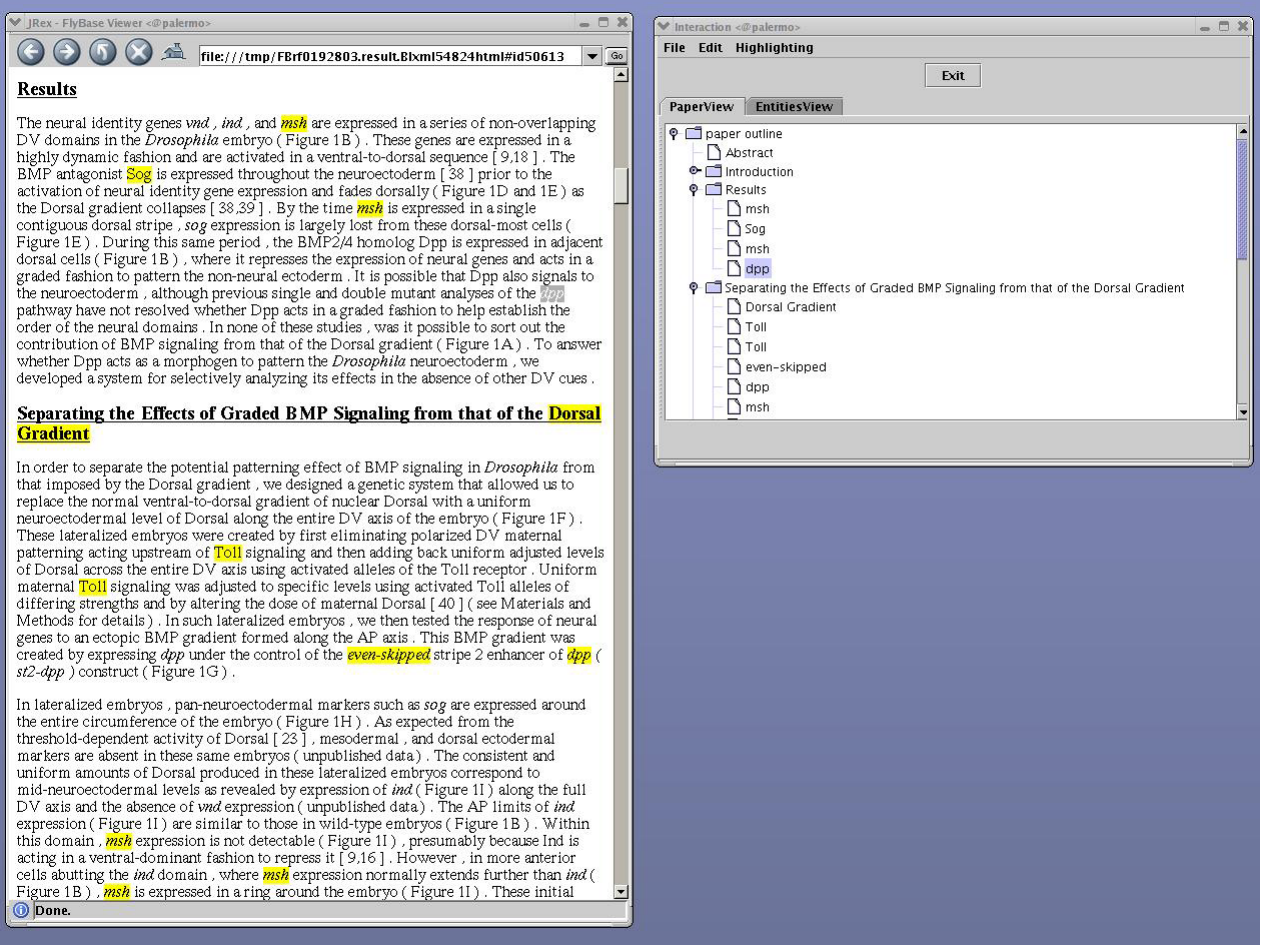
**PaperView navigator**. Screenshot of PaperBrowser's PaperView navigation mechanism, which lists automatically recognised gene names in the order in which they appear in each section.

**Figure 2:**
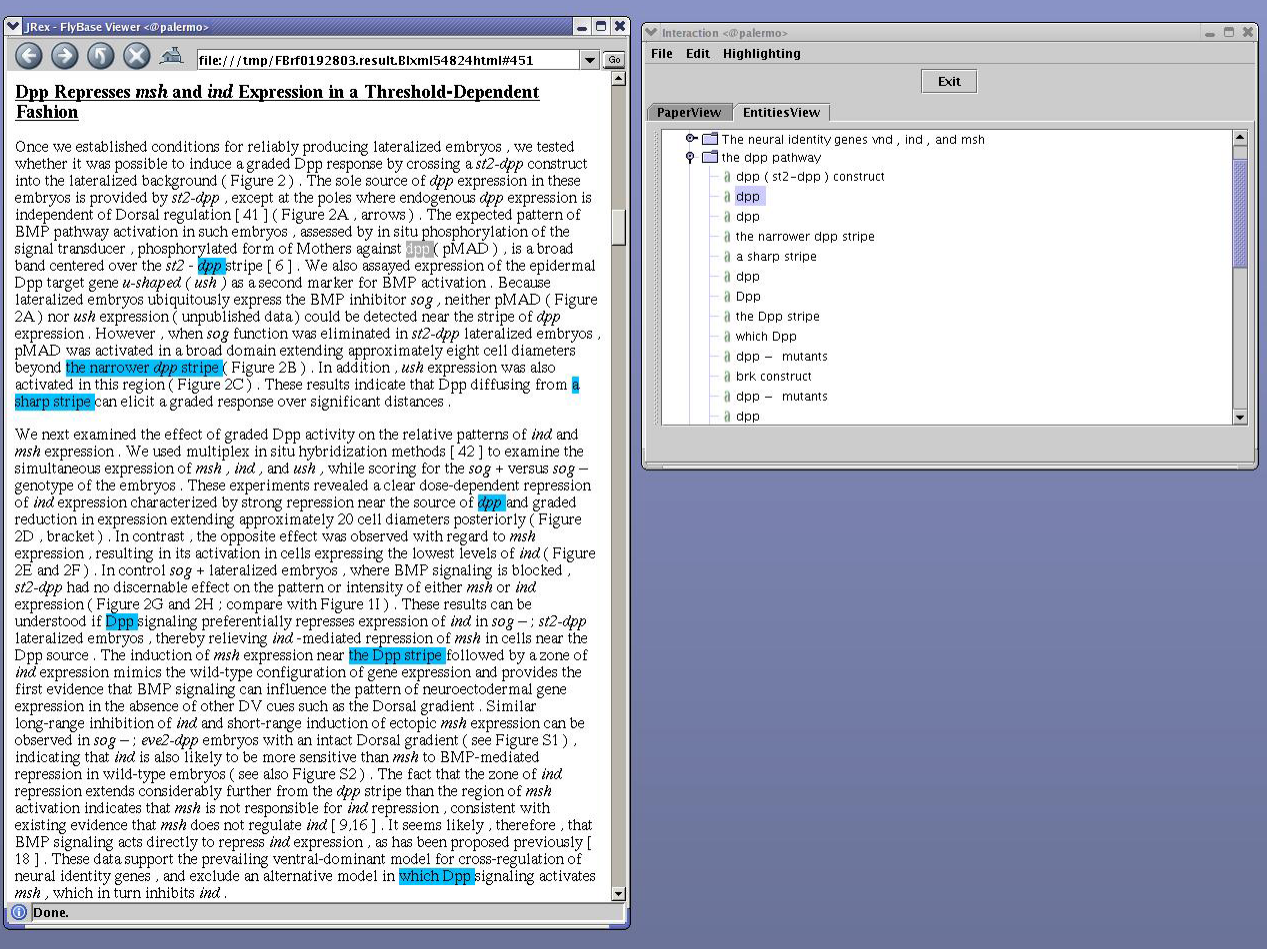
**EntitiesView navigator**. Screenshot of PaperBrowser's EntitiesView navigation mechanism, which groups together related noun phrases such as "dpp" and "the dpp pathway".

PaperBrowser is a web browser built on top of existing open source software with several additional functionalities aiming to improve the way in which the curator interacts with the article. To respond to the need to navigate the text more efficiently, PaperBrowser is equipped with two navigation mechanisms called PaperView and EntitiesView. These are organised in terms of the document sectioning and possible relations between groups of words (noun phrases), both of which are useful cues for curation as already mentioned. More specifically, PaperView lists gene names such as "dpp" or "Toll" in the order in which they appear in each section of the article (Figure 1). EntitiesView lists noun phrases related to the gene names such as "the dpp pathway" (Figure 2).

Clicking on a node in either PaperView or EntitiesView redirects PaperBrowser to the sentence that contains the corresponding gene name or noun phrase. When a name on PaperView is clicked, it is highlighted in a different colour (Figure 1). When EntitiesView is clicked, the gene name highlighting is switched off while all noun phrases listed together with the clicked node in EntitiesView are highlighted in the same colour (Figure 2). In this way the selected node and the related noun phrases become more visible in the text.

EntitiesView and PaperView are meant not only to provide the curator with an overview of the gene names and the related noun phrases in the article but also to support focused extraction of information, e.g. when the curator is looking for a gene name in a specific section or tries to locate a noun phrase referring to a certain gene product. They should also enable the curator to detect curatable regions and move back and forth between them quickly and efficiently.

### Natural Language Processing pipeline

**Figure 3:**
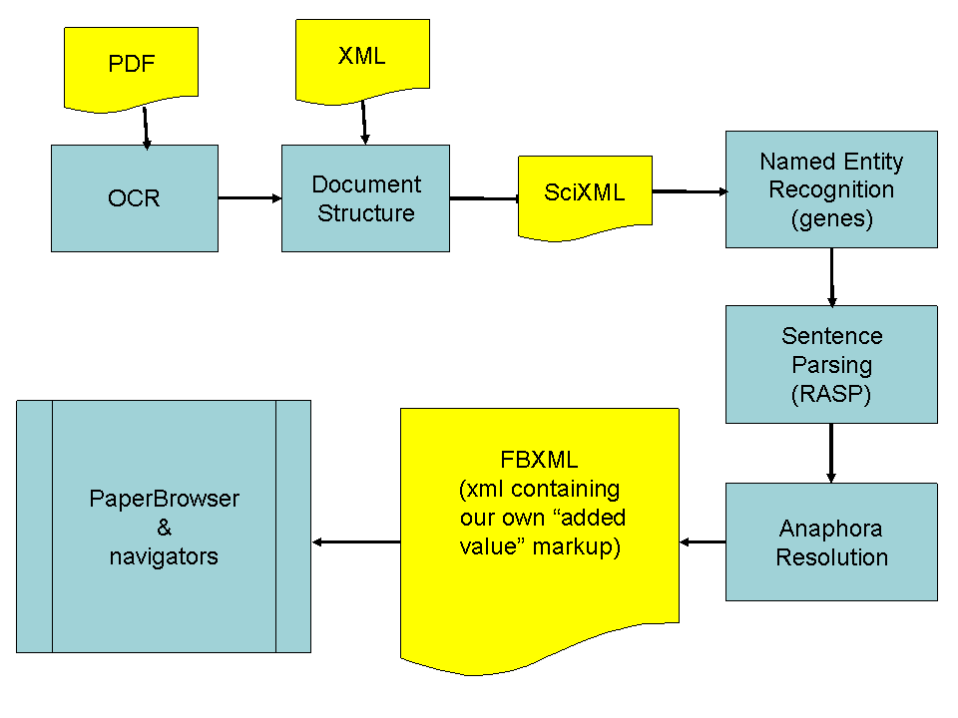
**Natural Language Processing pipeline**. Figure 3 shows how a series of NLP modules are arranged in a pipeline fashion to produce the format which is displayed on PaperBrowser for each curatable article.

PaperBrowser's backbone is the NLP pipeline which is shown in Figure 3. The pipeline can be fed with articles in XML format which are available from certain publishers. For articles which appear in PDF format only, a third-party commercial software for optical character recognition (OCR) is used for the initial PDF-to-XML translation. The Document Structure module translates the XML which is derived from the publisher or the OCR process into SciXML, a generic XML scheme defined to represent scientific articles [16]. At this stage, the different sections of the document as well as their headings and subheadings are recognised as explained in Lewin [17].

This output is then fed to a module for NER that implements machine learning to identify gene names in the text. NER can be performed either by using a Hidden Markov Model (HMM) or Conditional Random Fields (CRFs) as discussed by Vlachos [18]. Then, the RASP parser [19] is employed to identify the boundaries of each noun phrase (NP), its subconstituents and its grammatical relations with other constituents in the text. Each NP is classified semantically using information derived from the Sequence Ontology [20]. NER, parsing and semantic class information are then used to resolve the anaphoric dependencies between NPs as described in Gasperin [21].

A version of the article in FBXML (i.e. our customised XML format) which encodes the outputs of each process is displayed by PaperBrowser. The PaperView navigator makes use of the output of the NER system and information about the structure of the article, while EntitiesView utilises the output of the Anaphora Resolution module as well.

Following the standard evaluation methodology in NLP, the NER and the Anaphora Resolution modules were found to achieve state-of-the-art performance on full papers (Recall: 61.4%, Precision: 89.2%, F1: 72.7% for CRF-based NER [18]; Recall: 53.4%, Precision: 63.0%, F1:57.8% for Anaphora Resolution [21]). However, in order to appraise whether this performance can facilitate curation, these modules need to be embedded in an interface tailored to the user's needs [6]. Since evaluating these applications is time consuming and hard to perform in a large scale, most existing text mining systems used by curators have not been subjected to extensive user-based evaluations. The rest of the paper discusses how we assessed PaperBrowser's navigational functionalities by applying evaluation criteria of HCI.

## Results

In our previous study [10], we measured the time it took a curator to complete a curation record. The study revealed a trend for records to be completed faster by about 20% when the curator was interacting with the article via PaperBrowser as opposed to extracting information from a hard copy.

In this study, we focus on assessing whether PaperBrowser enhances the way in which the curators navigate an article. Since curators routinely use "Find" to navigate the electronic version of an article, we compare this mechanism with PaperBrowser's navigational aids. The text highlighting task, which is an integral part of curation as explained earlier, was used to collect data about how the curators navigate the text.

## Methods

PaperBrowser was updated to enable the curators to highlight text. *Highlighting events *and *navigation actions *(i.e. clicks on navigators and searches using "Find") were logged. PaperBrowser was also adjusted to enable loading an article without making its navigational functionalities available. This is similar to viewing the article on a standard browser.

Two curators participated in the study, one with low highlighting density and another with high highlighting density as identified during our observations. Both participants have more than two years curation experience and have used PaperBrowser before. The curators looked at issues from two open-access journals published since 2005 and identified 30 uncurated articles by applying their standard criteria. The articles contain various types of curatable information and were selected without any special adjustment to PaperBrowser. Each article was processed by the NLP pipeline, using the the publisher's XML as the input. The CRF-based system, which was shown to perform better than the HMM-based one in full-articles [18], was used for NER.

The curators were asked to interact with PaperBrowser and first identify curatable entities (genes and alleles). Then, they highlighted text that contains curatable information for each entity. To make the task as clear as possible, the curators were given step-by-step guidelines and examples of the kinds of curatable information that typically occur in an article. The guidelines and the examples are available in Additional File 1.

**Figure 4:**
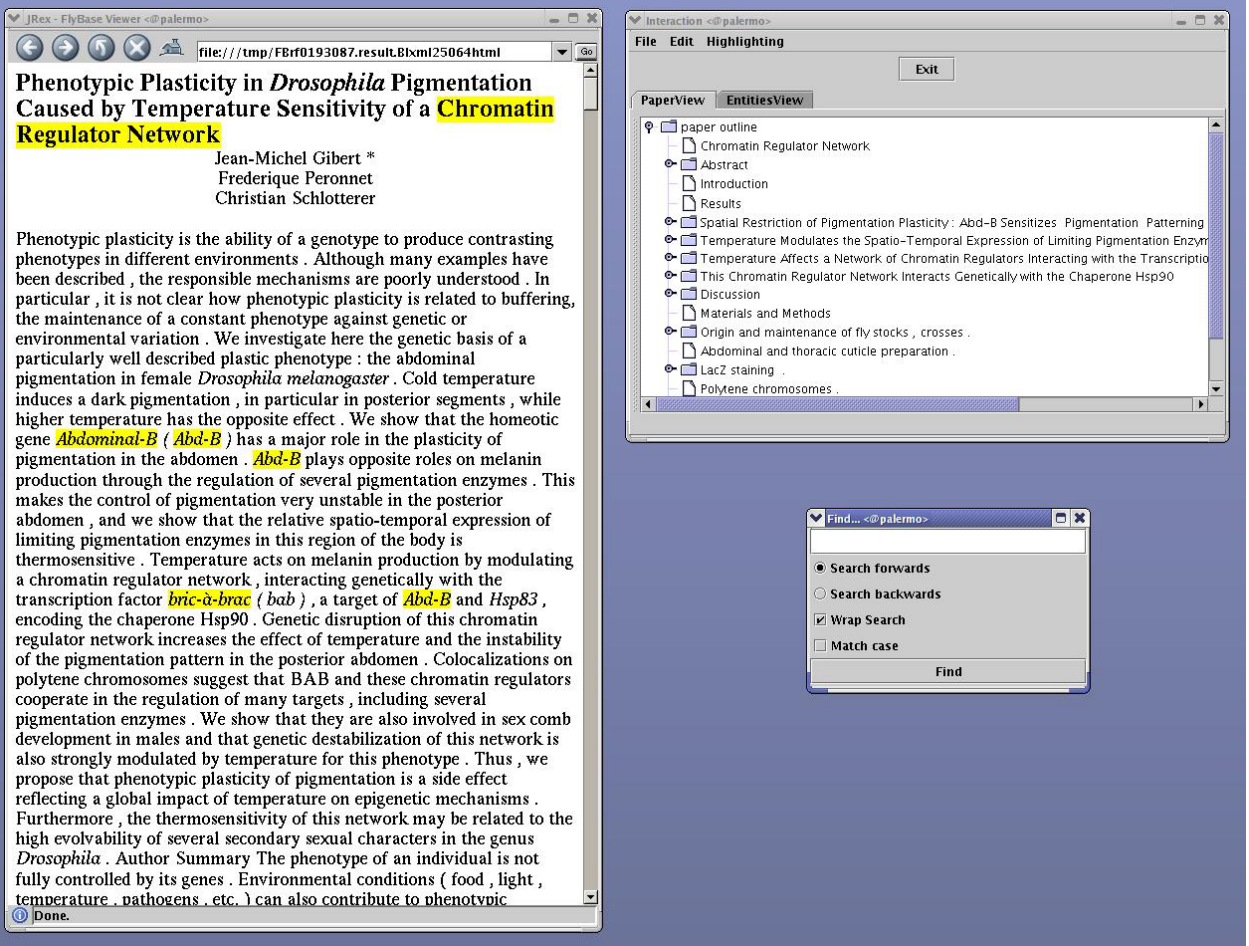
**Experimental condition**. Screenshot showing how PaperBrowser's windows were arranged in the experimental condition.

**Figure 5:**
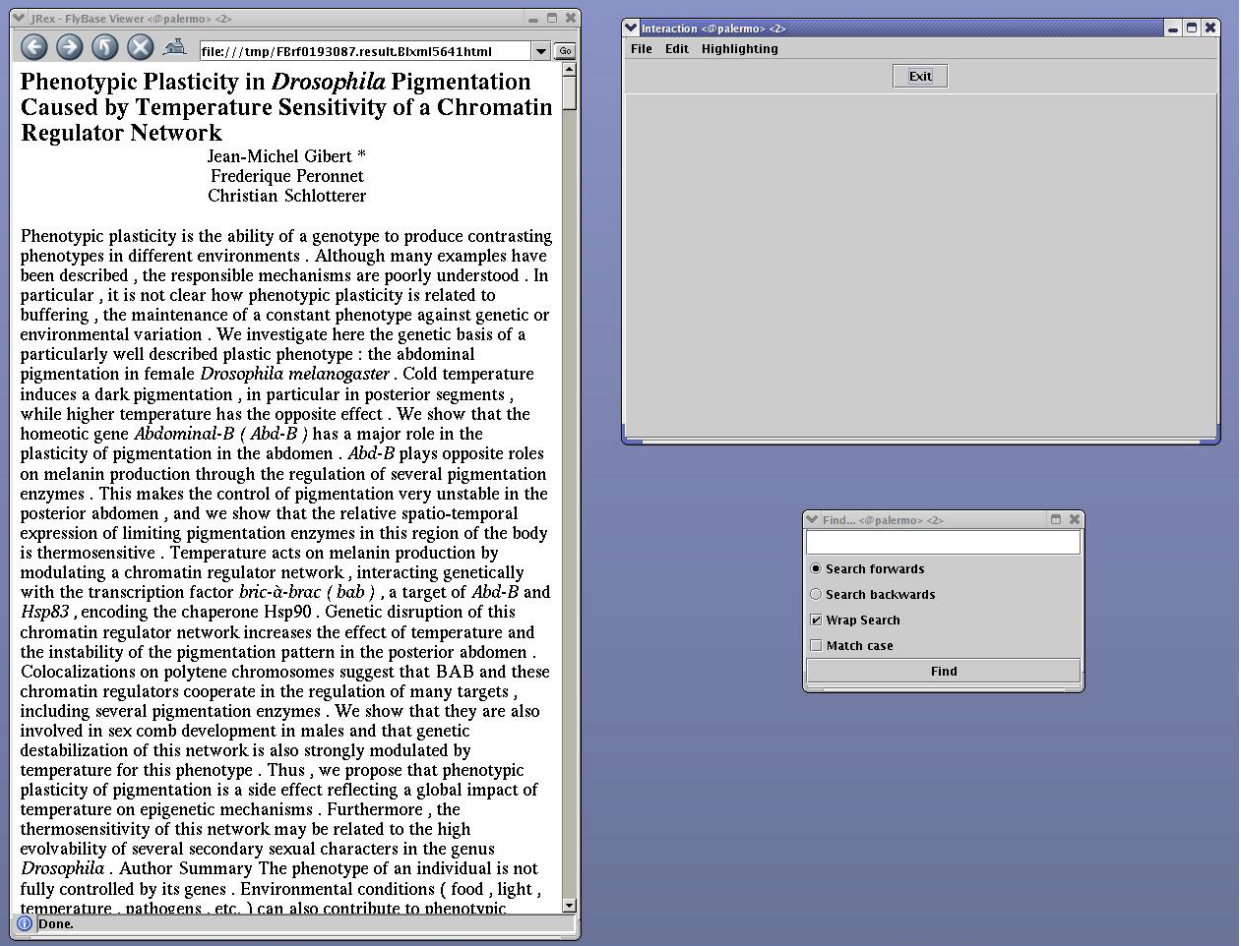
**Control condition**. Screenshot showing how PaperBrowser's windows were arranged in the control condition.

PaperBrowser's navigational functionalities were available for half of the articles (experimental condition). For the other half, these functionalities were not available (control condition). The "Find" function was always available. The presentation order was randomised and the assignment of articles to each condition was counterbalanced (so that both curators would see every article but the same article would be viewed by each curator in a different condition). By experimenting with a relatively large number of articles and by controlling the presentation mode, we believe to have overcome any possible selection bias caused by curators favouring certain types of articles. The same type of screen was used by both curators, who were instructed to arrange the windows as shown in Figure 4 (experimental condition) and Figure 5 (control condition). This arrangement remained constant throughout the study.

### Data analysis

Each of the 1655 datapoints (highlighting events) was classified as follows:

• Whether PaperBrowser's navigators were available when the article was viewed (NAVS:ON) or not (NAVS:OFF).

• Which curator did the highlighting (CURID:CUR01 or CURID:CUR02).

• Whether the navigators (PREV:NAVS), "Find" (PREV:FIND) or a combination of the two (PREV:BOTH) were used between two subsequent highlighting events. *Navigated events *are events immediately preceded by at least one navigation action. *Unnavigated events *were not immediately preceded by any navigation action and are labelled as PREV:NONE. Using the slider bar falls in this category too.

• Whether the text marked in a highlighting event followed (TEXT:AFTER) or preceded (TEXT:BEFORE) the text that had been highlighted in the immediately previous event. TEXT:BEFORE events represent cases of *highlighting-back *in the article.

• We also measured the distance in tokens (as recognised by the NLP analysis) between text regions marked by two subsequent highlighting actions, a variable that we call DIST. DIST was used to account for unnavigated events as explained below.

### Experimental questions

The study is designed to investigate the following questions:

• Do PaperBrowser's navigators provide the curators with an improved way to navigate the text compared to the navigation mechanism that was available to them previously (i.e. the "Find" function)?

• When are the navigators not used?

With respect to the second question, during our observations we noticed that navigation takes place to highlight segments which are far away from each other in the text. Hence, we expect DIST for navigated events to be significantly higher than DIST for unnavigated events.

The control condition provides evidence about how frequently "Find" is normally used. If "Find" is used much less frequently in the experimental condition and if PaperBrowser's navigators account for most of the navigated events in this condition, we can conclude that they have replaced "Find" as a mechanism for navigating the text.

Moreover, we compare PaperBrowser's navigators with "Find" by applying to the text highlighting task two evaluation criteria from HCI, namely *efficiency *and *utility *[11]. To estimate the efficiency of each navigation mechanism, we counted the number of navigation actions that preceded each navigated event. The fewer the actions, the more efficiently the curator accesses information.

To estimate the utility of each navigation mechanism, we measured the number of unnavigated events that followed each navigated event. The larger this count, the less the curators have to "flip back-and-forth" in the article. We also investigate whether our measure of utility is affected by the differences in highlighting density between the curators which were discussed earlier in the paper.

### Use of navigation mechanisms

As Table 1 shows, when the navigators were not available (NAVS:OFF), the "Find" function was used 45% of the time. Moreover, 69% of highlighting-back events (TEXT:BEFORE) were preceded by using "Find". These results indicate that the curators used "Find" to navigate the articles quite frequently in the control condition, especially when they were highlighting-back.

**Table 1:** Navigation using "Find"

Event type	NAVS:OFF	NAVS:ON
All highlighting events	45.02% (375/833)	3.16% (26/822)
TEXT:BEFORE	69.07% (67/97)	10.99% (10/91)

Table 1 shows the percentage of highlighting events which were navigated using "Find" (PREV:FIND) in control (NAVS:OFF) and experimental (NAVS:ON) conditions. The "All highlighting events" row shows the percentage of navigated events over all highlighting events. The TEXT:BEFORE row shows the percentage of navigated events when text was highlighted-back in the article.

However, when the navigators were available (Table 1, NAVS:ON), just 3% of the highlighting events were preceded by using "Find". Notably, only 2% (18/822) of the highlighting events in NAVS:ON were preceded by a combined use of both navigation mechanisms (PREV:BOTH). Moreover, PREV:FIND accounted for just 11% of highlighting-back events in NAVS:ON.

The curators preferred to use PaperBrowser's navigators over "Find" when the navigators were available.

As Table 2 shows, the curators only used PaperBrowser's navigators (PREV:NAVS) before 83% of the navigated events in NAVS:ON. Moreover, 86% of the navigated events in TEXT:BEFORE were also preceded by use of the navigators only. These results indicate that the curators identified curatable text without resorting to "Find" in the large majority of the navigated events in the experimental condition, including most instances of highlighting-back. In other words, PaperBrowser's navigators have replaced "Find" as a mechanism for navigating the article.

**Table 2:** Navigation preferences

Event type	PREV:FIND	PREV:NAVS
All navigated events	10.04% (26/259)	83.01% (215/259)
TEXT:BEFORE	13.70% (10/73)	86.30% (63/73)

Table 2 shows the percentage of navigated events using "Find" (PREV:FIND) and PaperBrowser's navigators (PREV:NAVS) in the experimental condition (NAVS:ON). The "All navigated events" row reports the percentage over all navigated events. The TEXT:BEFORE row shows the percentage over the navigated events in which text was highlighted-back in the article.

### Navigation distance

As Table 3 shows, mean DIST is much higher for navigated (PREV:FIND) than unnavigated (PREV:NONE) events in the control condition (NAVS:OFF). To test whether this difference is significant and whether the result holds for both curators, we conducted a 2 × 2 ANOVA followed by planned pairwise comparisons using the independent-samples two-tailed T-test as suggested in [22]. This procedure was followed for the statistical analyses reported in subsequent sections too. The "R" statistical software was used to run the tests [23].

**Table 3:** Mean distance in control condition

	PREV:FIND	PREV:NONE
Total mean	**1760.20**	**595.01**
Counts	375	458
Standard deviation	2476.82	1591.97
CUR01 mean	**1679.64**	**189.55**
Counts	334	201
Standard deviation	2507.45	660.43
CUR02 mean	**2416.51**	**912.13**
Counts	41	257
Standard deviation	2126.30	1988.33

Mean distance in tokens (DIST) between text marked in a navigated event (PREV:FIND) or an unnavigated event (PREV:NONE) and the text highlighted in the immediately preceding event in the control condition (NAVS:OFF). Curator-specific data are indicated by the curator's ID (CUR01 vs CUR02). Mean is calculated as the sum of all observations divided by the number of observations (counts).

More specifically, a 2 × 2 ANOVA with conditions PREV (FIND vs NONE) and CURID (CUR01 vs CUR02) showed a significant main effect for PREV (F(1,829)<88.123, p < 0.001). The effect of CURID (F(1,829)< 0.001, p = 0.996) and the interaction between PREV and CURID (F(1,829)< 0.001, p = 0.970) were not significant. These results show that the text marked between two subsequent highlighting events tends to be much further away when "Find" is used compared to the cases in which "Find" is not used. Two-tailed T-tests on PREV (FIND vs NONE) for each curator (CUR01: t(533) = 8.252, p < 0.001; CUR02: t(296) = 4.456, p < 0.001) confirmed that this result holds for both curators.

Table 4 shows that mean DIST is much higher for navigated (PREV:NAVS) than unnavigated (PREV:NONE) events in the experimental condition (NAVS:ON) too. A 2 × 2 ANOVA with conditions PREV (NAVS vs NONE) and CURID (CUR01 vs CUR02) showed a significant main effect for PREV (F(1,774) = 407.830, p < 0.001) and no significant main effect for CURID (F(1,774) = 1.176, p = 0.279). The interaction between PREV and CURID was also significant (F(1,774) = 47.223, p < 0.001). As [22] discuss, the significant interaction means that the effect of PREV is more pronounced for one of the curators. This is also evident by the high ratio (1.99) of the difference in mean DIST (PREV:NAVS minus PREV:NONE) for CUR02 over the same difference for CUR01.

**Table 4:** Mean distance in experimental condition

	PREV:NAVS	PREV:NONE
Total mean	**2842.76**	**333.57**
Counts	215	563
Standard deviation	2828.91	1012.22
CUR01 mean	**2406.74**	**105.98**
Counts	180	243
Standard deviation	2624.99	174.58
CUR02 mean	**5085.14**	**506.39**
Counts	35	320
Standard deviation	2812.57	1308.64

Mean distance in tokens (DIST) between text marked in a navigated event (PREV:NAVS) or an unnavigated event (PREV:NONE) and the text highlighted in the immediately preceding event in the experimental condition (NAVS:ON). Curator-specific data are indicated by the curator's ID (CUR01 vs CUR02).

These results show that the text marked between two subsequent highlighting events tends to be much further away when PaperBrowsers' navigators are used compared to the cases in which they are not used.

Two-tailed T-tests on PREV (NAVS vs NONE) for each curator (CUR01: t(421) = 13.628, p < 0.001; CUR02: t(353) = 16.923, p < 0.001) further confirmed that this finding holds for both curators.

In summary, the results in this section confirm our prediction that DIST for navigated events is significantly higher than DIST for unnavigated events. This accords with our account for unnavigated events: When the highlighted segments of text appear close to each other, the curators refrain from navigating the article. Navigation is more likely to occur to highlight segments which appear further away from each other in the article.

### Navigation efficiency

As Table 5 shows, the curators highlighted curatable text after just 3 navigation actions when the navigators were available (PREV:NAVS in NAVS:ON). This represents a relative improvement on efficiency by 58.22% compared to the average number of preceding "Find" actions in the control condition (PREV:FIND in NAVS:OFF).

**Table 5:** Navigation efficiency

	PREV:FIND	PREV:NAVS
Total mean	**7.63**	**3.19**
Counts	375	215
Standard deviation	9.31	2.83
CUR01 mean	**7.57**	**3.31**
Counts	334	180
Standard deviation	9.24	2.97
CUR02 mean	**8.22**	**2.49**
Counts	41	35
Standard deviation	9.93	1.85

Mean number of navigation actions occurring before each navigated event using "Find" (PREV:FIND) in the control condition and PaperBrowser's navigators (PREV:NAVS) in the experimental condition. Curator-specific data are indicated by the curator's ID (CUR01 vs CUR02).

A 2 × 2 ANOVA with conditions PREV (FIND vs NAVS) and CURID (CUR01 vs CUR02) showed a significant main effect for PREV (F(1, 586) = 46.695, p < 0.001), no main effect of CURID (F(1, 586) = 0.2834, p = 0.595), and no interaction between PREV and CURID (F(1, 586) = 0.602, p = 0.438). Two-tailed T-tests on PREV (FIND vs NAVS) for each curator (CUR01: t(512) = 6.027, p < 0.001; CUR02: t(74) = 3.364, p = 0.001) further confirmed that these findings hold for both curators. These results show that it takes significantly fewer user actions to identify curatable text when PaperBrowser's navigators are used in comparison to navigating the article using "Find".

### Navigation utility

As Table 6 shows, when the navigators were available (PREV:NAVS in NAVS:ON), the average number of unnavigated events that followed each navigated event was increased by 74.42% compared to the mean count of unnavigated events that follow a "Find" action in the control condition (PREV:FIND in NAVS:OFF).

**Table 6:** Navigation utility

	PREV:FIND	PREV:NAVS
Total mean	**1.63**	**2.84**
Counts	375	215
Standard deviation	2.35	4.17
CUR01 mean	**1.37**	**2.23**
Counts	334	180
Standard deviation	1.29	1.95
CUR02 mean	**3.20**	**6.57**
Counts	41	35
Standard deviation	5.90	8.54

Mean number of unnavigated events occurring after an event navigated using "Find" (PREV:FIND) in the control condition or PaperBrowser's navigators (PREV:NAVS) in the experimental condition. Curator-specific data are indicated by the curator's ID (CUR01 vs CUR02).

A 2 × 2 ANOVA with conditions PREV (FIND vs NAVS) and CURID (CUR01 vs CUR02) showed a significant main effect for PREV (F(1,586) = 23.085, p < 0.001). Two-tailed T-tests on PREV (FIND vs NAVS) for each curator (CUR01: t(512) = -6.048, p < 0.001; CUR02: t(74) = -2.028, p < 0.05) further confirmed that these findings hold for both curators. This result shows that using PaperBrowser's navigators significantly reduces unnecessary "back and forth flipping" in the article.

The effect of CURID was also significant (F(1,586) = 72.138, p < 0.001). This is due to the differences in highlighting density between the curators. The highlighting density of CUR02 is higher than that of CUR01, thus giving rise to a significantly higher number of highlighting events in each condition. The interaction between PREV and CURID was significant too (F(1,586) = 11.741, p < 0.001). This means that the effect for PREV is more pronounced for one of the curators. This is also evident by the high ratio (3.89) of the difference in mean utility (PREV:FIND minus PREV:NAVS) for CUR02 over the same difference for CUR01.

Taken together, these results suggest that the significant improvement in utility occurs irrespective of the different ways in which the curators highlight text in the article.

## Discussion

The usability of systems developed to address the information overload faced by experts in life sciences has begun to be explored only very recently [8]. Alex et al. [9], whose work is the closest to ours, report on three experiments measuring whether curation can be speeded up using NLP. Although curation appears to be faster sometimes in their experiments, they conclude that a careful consideration of several other factors is required before a verdict on the usefulness of NLP for curation is reached.

In this paper, we extended our previous study on measuring curation time by using additional HCI criteria to evaluate the usefulness of PaperBrowser. While Alex et al. do not discuss whether users were involved in the development of their curation interface, PaperBrowser was developed under a user-centered approach. Although this adds to the time and complexity of the development process, it increases the likelihood of producing a useful system [11].

Among the several engines developed to enhance literature search, only the system of Hearst et al. [7] is reported to have been developed and evaluated using HCI principles. However, like most other information retrieval systems, it only employs shallow techniques for text analysis. By contrast, PaperBrowser indexes an article using the output of several advanced NLP modules.

Given that PaperBrowser navigators make use of the NLP output, assessing its navigational capabilities provides new evidence in favour of the usefulness of NLP for curation. Our results indicate that PaperBrowser's navigators essentially replaced "Find" as a mechanism for navigating the article. Using PaperBrowser's navigators leads to a significant improvement on efficiency of about 58% (at the 99.9% confidence level) for both curators compared to navigating the article using "Find". A significant improvement on utility of about 74% at the same confidence level for both curators, irrespective of their differences in highlighting density, was also observed. To date, over 75 articles have been curated with the use of PaperBrowser without aborting it (100% success rate), further suggesting that PaperBrowser can support FlyBase curation successfully.

The DIST variable was used to account for unnavigated events. DIST for navigated events was found to be significantly higher than DIST for unnavigated events in both conditions, as expected from our observations. In other words, highlighting a segment which is far away from the text that had been marked in the immediately previous highlighting event is likely to be preceded by navigation. By contrast, both curators refrained from performing a navigation action when they were highlighting excerpts closer to each other. To the best of our knowledge, this is the first attempt to describe how curators use an NLP-powered interface for a task integral to curation on the basis of empirical data.

To run the reported study, we used articles in the publisher's XML format as the input to the NLP pipeline, thus ignoring the noise that is introduced by the OCR process when this input is not available. We intend to address this limitation in our future studies.

Investigating curation accuracy and agreement between curators was left outside the scope of this study to avoid making it too complicated. We assumed that the curators always identify relevant information when they highlight text, which is reasonable given the extensive training that they undergo. Extending our evaluation framework to investigate these additional variables constitutes another direction for future work.

## Conclusion

That NLP can be a useful aid for curation has long been assumed within the biomedical text mining community. However, there has been little evidence so far that this is indeed the case. How to utilise this technology within an existing curation workflow has been equally unclear.

PaperBrowser is an NLP-engined curation interface developed under a user-centered approach and aiming to enhance the way in which the curator navigates an article. This paper discusses how HCI principles have been applied to develop and evaluate PaperBrowser, providing the text mining community with a general and replicable framework.

Our evaluation study shows that PaperBrowser improves navigational efficiency and provides FlyBase curators with enhanced utility compared to the navigation mechanism that has been available to them previously. We conclude that state-of-the-art performance in NER and Anaphora Resolution can be combined with the navigational functionalities of PaperBrowser to support FlyBase curation quite successfully. Given the large number of groups which curate in a similar way as FlyBase, this study is likely to have far-reaching implications.

## Availability and Requirements

• **Project name**: FlySlip

• **Project website**: 

• **Programming language**: Java 1.4.2 or above.

• **Restrictions**:

PaperBrowser is implemented on top of Mozilla Gecko and JREX. It runs on 32-bit Linux Fedora Core 3 and is freely available for non-commercial use from the Resources section of the FlySlip website.

The NLP pipeline uses commercial software for OCR and the PTX reformatter for the initial XML output [24], for which licences are required, and the RASP toolkit and project specific software for NER and Anaphora Resolution, which are freely distributed under a non-commercial licence.

## Authors' contributions

NK, RS and PM designed and ran the evaluation study. NK also carried out the curation observations, performed the statistical analyses and drafted the manuscript. IL designed and implemented PaperBrowser. AV and CG implemented the NLP modules for NER and Anaphora Resolution respectively. TB contributed to the development of the pipeline and led all aforementioned efforts as the project's PI together with RD. All authors read and approved the final manuscript.

## Supplementary Material

Additional file 1 **Step-by-Step Guide for Highlighting**. Guidelines given to the curators for the text highlighting task. Click here for file
