# Supplementary material for: Natural Language Processing in aid of FlyBase curators
Source: BMC Bioinformatics. 2008 Apr 14;9:193. doi: 10.1186/1471-2105-9-193 (PMC2375127; doi:10.1186/1471-2105-9-193)
Supplement: Additional file 1 — Step-by-Step Guide for Highlighting. Guidelines given to the curators for the text highlighting task. [file 1471-2105-9-193-S1.pdf]

Step-by-Step Guide for Highlighting

Abstract

The following step-by-step guidelines for text highlighting were given to FlyBase curators. The text highlighting task was part of a study assessing the navigational functionalities of PaperBrowser. The results of the study are reported in Karamanis at al. (2008) *Natural Language Processing in Aid of FlyBase Curators*, BMC Bioinformatics.

This is a step-by-step guide of how the curator should approach the highlighting task.

First, take around 2 minutes to explore the layout of the paper. Read the abstract to see if you can get an overview of what the paper is about and look to see where the different sections start and end, such as Results, Methods etc.

Second, highlight all gene and allele names in the abstract, results, materials and methods and figure legends sections. Try not to highlight the same name more than once. During this part of the task, try to get an idea of which genes have sufficient amounts of data associated with them in the paper. Use these genes to begin with to perform the next part of the task.

In general, you should start looking for data associated with any gene that is central to the paper. Usually, such genes will be mentioned in the abstract. After finding text associated with these genes, move on to trying to find data for other genes until you think you have fully answered all the questions below.

Now, taking one gene at a time, try to highlight text that answers the following questions:

Is there any expression data for this gene? Highlight text which would provide evidence of gene expression study. Some papers are about nothing but expression, so the curator should not try to find every single expression-related sentence as this is outside the remit of Cambridge curation. They should choose one or two sentences per gene for this.

Is there any text that describes "molecular modifications" of alleles? This includes any text that describes constructs, aa replacements, mutagens etc. These sentences will be either in the materials and methods section or in the results section.

Is there any text that describes allele phenotypes? These sentences should be in the results section or in figure legends.

Is there any text that is appropriate to use for finding GO terms or for recording sentences that describe the role of the gene as free text? Don't be tempted to look in the discussion for this text. These sentences may overlap phenotype expression text that we have already highlighted.

Below are examples of the type of sentences that would answer each one of these questions.

Examples of expression sentences:

- HA-tagged forms of Dally and Dally-GAG expressed in S2 cells were isolated from DEAE Sepharose by stepwise elution with increasing concentrations of NaCl and analyzed by Western blotting using HA.11 antibody.[1]
- For these experiments, Myc-tagged Dally or Dally-GAG was co-expressed in S2 cells with HA-tagged Dpp, and Dpp-bound proteins were recovered by immunoprecipitation with anti-HA antibody.[1]
- A late third instar wing disc expressing UAS-DN-DBT with act>CD2>Gal4 was immunostained to show Ci155 accumulation (detected by the Ci antibody 2A) and CD2 expression.[2]
- Cells expressing CRL at reduced levels accumulate Ci155 but do not ectopically express dpp-lacZ.[2]
- P compartment smo3 cells expressing HA-CiDSGX2 no longer inhibited hh-lacZ expression when PKA activity was blocked by coexpressing R*h.[2]

These sentences should be the kind that are of interest to expression curation, so we don't want sentences such as "Coexpressing DBT/CKIe or CKIa rescued Ci processing defects caused by expressing CRL or CRS" because this sentence does not represent expression data.

Examples of molecular modification sentences:

- In DN-DBT/CKIe, K38 was substituted to R by PCR-based site-directed mutagenesis.[2]
- CKI isoforms were tagged with a Flag epitope at their N-termini and subcloned into the pUAST vector.[2]
- A 540 bp fragment of dEset cDNA was PCR amplified with the 12196UBam (5'-CAAGGATCCTGTCTGGCCGAGTACTCCAT) and 12196LKpn (5'-TGCGGTACCTGATAGTTTGGCCGGAAGTC) primers by using the pHIBS and pUdsGFP vectors as described by (NAGEL et al. 2002).[3]
- pPUAST constructs containing cDNAs fused to the EGFP coding sequence were used for transfection of Drosophila S2R+ cells.[4]

Examples of phenotype sentences:

- Unexpectedly, we found that anteriorly situated clones expressing DN-DBT often induced wing duplications.[2]
- dcole88 mutants die at late embryonic and early larval stages.[2]
- Hemizygotes with Nuf2SH2276 over Df(2L)ade3, which deletes Nuf2, were found to die during the late pupal stages, i.e., earlier than Nuf2SH2276 homozygotes.[5]

- Compared to sibling control larvae, the mutants had only rudimentary imaginal discs and small brains, suggesting that Spc25 is required in mitotically proliferating cells.[5]
- Overexpressing wildtype Dally using ey-Gal4 caused an overgrowth phenotype as it had in the wing.[1]

Examples of GO/biological role sentences:

- We demonstrate that Drosophila dSet2 encodes a developmentally essential histone H3 lysine 36 (K36) methyltransferase.[3]
- Loss and gain of function studies demonstrate that pcs is necessary at distinct times for muscle specification and morphogenesis.[5]
- Subcellular organelle fractionation and confocal microscopy of Drosophila S2 cells confirmed that the immunoreactive 25 kDa protein is present in mitochondria but not in the cytosol.[6]
- Proper expression of HSP70 and HSP23 expression was dependent on menin, defining a new function for this protein and indicating that menin is a key regulator of the stress response in Drosophila melanogaster.[7]

The example sentences come from the following papers:

1) Kirkpatrick et al., 2006, Dev. Biol. 300(2): 570-582.
2) Jia et al., 2005, Dev. Cell 9(6): 819-830.
3) Stabell et al., 2007, Biochem. Biophys. Res. Commun. 359(3): 784-789.
4) Schittenhelm et al., 2007, Chromosoma 116(4): 385-402.
5) Beckett and Baylies, 2006, Dev. Biol. 299(1): 176-192.
6) Liu et al., 2007, Insect Biochem. Molec. Biol. 37(2): 155-163.
7) Papaconstantinou et al., 2005, Molec. Cell. Biol. 25(22): 9960-9972.
